# Icariin, a Novel Blocker of Sodium and Calcium Channels, Eliminates Early and Delayed Afterdepolarizations, As Well As Triggered Activity, in Rabbit Cardiomyocytes

**DOI:** 10.3389/fphys.2017.00342

**Published:** 2017-05-29

**Authors:** Wanzhen Jiang, Mengliu Zeng, Zhenzhen Cao, Zhipei Liu, Jie Hao, Peipei Zhang, Youjia Tian, Peihua Zhang, Jihua Ma

**Affiliations:** Cardio-Electrophysiological Research Laboratory, Medical College, Wuhan University of Science and TechnologyHubei, China

**Keywords:** icariin, antiarrhythmic drug, ion currents, action potential, cardiomyocytes

## Abstract

Icariin, a flavonoid monomer from *Herba Epimedii*, has confirmed pharmacological and biological effects. However, its effects on arrhythmias and cardiac electrophysiology remain unclear. Here we investigate the effects of icariin on ion currents and action potentials (APs) in the rabbit myocardium. Furthermore, the effects of icariin on aconitine-induced arrhythmias were assessed in whole rabbits. Ion currents and APs were recorded in voltage-clamp and current-clamp mode in rabbit left ventricular myocytes (LVMs) and left atrial myocytes (LAMs), respectively. Icariin significantly shortened action potential durations (APDs) at 50 and 90% repolarization (APD_50_ and APD_90_) and reduced AP amplitude (APA) and the maximum upstroke velocity (V_max_) of APs in LAMs and LVMs; however, icariin had no effect on resting membrane potential (RMP) in these cells. Icariin decreased the rate-dependence of the APD and completely abolished anemonia toxin II (ATX-II)-induced early afterdepolarizations (EADs). Moreover, icariin significantly suppressed delayed afterdepolarizations (DADs) and triggered activities (TAs) elicited by isoproterenol (ISO, 1 μM) and high extracellular calcium concentrations ([Ca^2+^]_o_, 3.6 mM) in LVMs. Icariin also decreased I_NaT_ in a concentration-dependent manner in LAMs and LVMs, with IC_50_ values of 12.28 ± 0.29 μM (*n* = 8 cells/4 rabbits) and 11.83 ± 0.92 μM (*n* = 10 cells/6 rabbits; *p* > 0.05 vs. LAMs), respectively, and reversed ATX-II-induced I_NaL_ in a concentration-dependent manner in LVMs. Furthermore, icariin attenuated I_CaL_ in a dose-dependent manner in LVMs. The corresponding IC_50_ value was 4.78 ± 0.89 μM (*n* = 8 cells/4 rabbits), indicating that the aforementioned current in LVMs was 2.8-fold more sensitive to icariin than I_CaL_ in LAMs (13.43 ± 2.73 μM; *n* = 9 cells/5 rabbits). Icariin induced leftward shifts in the steady-state inactivation curves of I_NaT_ and I_CaL_ in LAMs and LVMs but did not have a significant effect on their activation processes. Moreover, icariin had no effects on I_K1_ and I_Kr_ in LVMs or I_to_ and I_Kur_ in LAMs. These results revealed for the first time that icariin is a multichannel blocker that affects I_NaT_, I_NaL_ and I_CaL_ in the myocardium and that the drug had significant inhibitory effects on aconitine-induced arrhythmias in whole rabbits. Therefore, icariin has potential as a class I and IV antiarrhythmic drug.

## Introduction

Icariin (C_33_H_40_O_15_, molecular weight = 676.7), the chemical structure of which has been reported by Tao et al. (2013), is a flavonoid monomer extracted from *Herba Epimedii*. It has been confirmed to have a variety of pharmacological and biological effects, including anti-inflammatory (Xu et al., 2010; Tao et al., 2013), antioxidant (Liu et al., 2004; Huang et al., 2014), anti-tumor (Wang et al., 2011; Tan et al., 2016), and neuroprotective effects (Liu et al., 2011). It was recently reported that icariin protected H9c2 cells from apoptosis by inhibiting endoplasmic reticular stress and the reactive oxygen species-dependent JNK and p38 pathways (Zhang et al., 2013; Zhou et al., 2014). Icariin was also found to ameliorate cardiac remodeling and left ventricular dysfunction in rats with heart failure by attenuating matrix metalloproteinase activity and myocardial apoptosis (Song et al., 2011). Furthermore, icariin protected the heart from ischemia-reperfusion injury through PI3K-Akt signaling pathway activation (Ke et al., 2015). Additionally, Sun et al. (2011) found that icariin facilitated the differentiation of mouse embryonic cells into cardiomyocytes. The results of these studies indicate that icariin has cardioprotective effects. However, the effects of icariin on APs and ion channels in cardiomyocytes have not been reported. Thus, the aim of the present study was to investigate the effects of icariin on action potentials (APs), ion currents in cardiomyocytes, as well as arrhythmias in whole rabbits, and to further investigate the medicinal value of icariin for the treatment of heart diseases.

## Materials and methods

### Cardiomyocyte isolation

The animal experiments performed in this investigation conformed to the Guide for Care and Use of Laboratory Animals of Hubei Province, China, and the study protocol was approved by Experimental Animal Ethics Committee of Wuhan University of Science and Technology. Hearts from adult New Zealand white rabbits (1.5–2 kg) of either sex were quickly removed and retrogradely perfused by the Langendorff method, as described previously (Wu, 2005), with Ca^2+^-free Tyrode solution containing the following compounds (in mM): 135 NaCl, 5.4 KCl, 1.0 MgCl_2_, 10 glucose, 0.33 NaH_2_PO_4_, and 10 HEPES, pH 7.4 with NaOH for 5 min. Then, hearts were perfused with Ca^2+^-free Tyrode solution containing collagenase type I (1 g/l) and bovine serum albumin (BSA, 1 g/l) for 30–40 min before being perfused with KB solution for another 5 min. After perfusion, the left ventricle and left atrium were isolated and gently agitated in KB solution. The cardiomyocytes were filtered through a nylon mesh and stored in KB solution containing the following compounds (in mM): 70 KOH, 40 KCl, 20 KH_2_PO4, 50 glutamic acid, 20 taurine, 0.5 EGTA, 10 glucose, 10 HEPES, and 3.0 MgSO4, pH 7.4 with KOH. All solutions used in this study were saturated with 95% O_2_ and 5% CO_2_ and were maintained at 37°C.

### AP recordings

For AP recording, quiescent and Ca^2+^-tolerant cardiomyocytes were bathed in standard Tyrode solution. The patch pipette solution contained the following reagents (in mM): 110 K-aspartate, 30 KCl, 5 NaCl, 10 HEPES, 0.1 EGTA, 5 MgATP, 5.0 creatine phosphate, and 0.05 CAMP, pH 7.2 with KOH. When filled with pipette solution, the electrode resistance was in the range of 1.5–2.5 MΩ. APs were induced in current-clamp mode by 1.5-fold diastolic threshold current pulses of 5 ms in duration at different pacing cycle lengths (CLs).

### Ion current recordings

Currents were recorded with a patch-clamp amplifier (EPC9, Heka electronic, Lambrecht, Pfalz, Germany) and were filtered at 2 kHz and digitized at 10 kHz.

The bath solution used for I_NaT_ recording contained the following compounds (in mM): 30 NaCl, 1.0 CaCl_2_, 105 CsCl, 1.0 MgCl_2_, 0.05 CdCl, 5.0 HEPES, and 5.0 glucose, pH 7.4 with CsOH, and 1 μM nicardipine was added to the bath solution to block I_CaL_. The pipette solution contained the following compounds (in mM): 120 CsCl, 1.0 CaCl_2_, 5.0 MgCl_2_, 5.0 Na_2_ATP, 10 TEA-Cl, 11 EGTA, and 10 HEPES, pH 7.3 with CsOH. I_NaT_ was determined by 300-ms depolarization pulses from −70 mV to +40 mV in 5-mV increments—using a holding potential (HP) of −90 mV—at 0.5 Hz. For the steady-state inactivation protocols, currents were recorded using 100-ms conditional prepulses from −100 mV to −50 mV in 5 mV increments—using a HP of −90 mV—followed by a 100-ms test pulse at −20 mV and 0.5 Hz.

The bath solution used for I_NaL_ recording contained the following compounds (in mM): 135 NaCl, 5.4 CsCl, 1.0 MgCl_2_, 10 glucose, 0.33 NaH_2_PO_4_, 0.3 BaCl_2_, 10 HEPES, and 1.8 CaCl_2_, pH 7.4 with NaOH, and 1 μM nicardipine was added to the bath solution to block I_CaL_. The pipette solution used for this experiment was the same as that used for I_NaT_ recording I_NaL_ was recorded using a 300-ms depolarization pulse at a HP of −90 mV, followed by pulses with potentials that were increased from −80 mV to +60 mV in 10-mV increments, and was measured at 200 ms in depolarization testing pulse.

The bath solution (except nicardipine) used for I_CaL_ recording was the same as that used for I_NaL_ recording. The electrode was filled with an internal solution containing the following compounds (in mM): 80 CsCl, 60 CsOH, 40 aspartate acid, 0.65 CaCl_2_,5.0 HEPES, 10 EGTA, 5.0 MgATP, and 5.0 Na_2_-creatine phosphate, pH 7.2 with CsOH. I_CaL_ was determined using 300-ms voltage steps with potentials that were increased from −40 mV to +50 mV in 5-mV increments at 0.5 Hz. For the steady-state inactivation protocol, I_CaL_ was determined using 2,000-ms conditional prepulses with potentials that were increased from −50 mV to 0 mV in 5-mV increments—using a HP of −40 mV—followed by a 300-ms test pulse at 0 mV.

For I_K1_ recording, the cells were bathed with Tyrode solution, and 1 μM nicardipine was used to block I_CaL_. The internal solution contained the following compounds (in mM): 140 KCl, 1.0 MgCl_2_, 5.0 K_2_ATP, 10 EGTA, and 5.0 HEPES, pH 7.3 with KOH.

The external solution used to record I_Kr_ contained the following compounds (in mM): 135 NaCl5.4 KCl, 1.0 MgCl_2_, 5.0 glucose, 0.2 CdCl_2_, 0.33 NaH_2_PO_4_, 5.0 HEPES, and 1.0 CaCl_2_, pH 7.4 with NaOH, and 30 μM chromanol 293B was used to block I_Ks_. The pipette solution contained the following compounds (in mM): 140 KCl, 1.0 MgCl_2_, 2.0 Na_2_ATP, 10 EGTA, and 5.0 HEPES, pH 7.25 with KOH.

The bath solution used to elicit I_to_ contained the following compounds (in mM): 140 NaCl, 5.4 KCl, 1.0 MgCl_2_, 10 glucose, 0.33 NaH_2_PO_4_, 5 HEPES, and 1.8 CaCl_2_, pH 7.4 with NaOH. The internal solution contained the following compounds (in mM): 110 K-aspartate, 20 KCl, 0.1 GTP, 1.0 MgCl_2_, 10 HEPES, 5.0 EGTA, 5.0 MgATP, and 5.0 creatine phosphate, pH 7.2 with KOH. BaCl_2_(200 μM), CdCl_2_ (200 μM), and atropine (1 μM) were used to block I_K1_, I_CaL_, and I_KAch_, respectively.

The bath solution and pipette solution used to record I_Kur_ were the same as those used to record I_to_, but the pulse protocol was different from that used to record I_to_ (see the Results Section).

### Aconitine-induced arrhythmias in whole rabbits

Twenty healthy New Zealand rabbits were randomly divided into two groups (*n* = 10 for each group): normal saline (NS) and icariin. In the NS group, saline was injected intraperitoneally within half an hour before the experiment. In the icariin group, 3 mg/kg icariin was injected intraperitoneally within half an hour before the experiment. At the beginning of the experiments, both groups of rabbits were anesthetized with xylazine (7.5 mg/kg, i.m.) and ketamine (30 mg/kg, i.v.) through ear vein injection. A standard limb lead II electrocardiogram (ECG) was recorded using the BL-420F data acquisition and analysis system (Chengdu TaiMeng, Sichuan, China) for 120 min following the application of 2 μg/kg/min aconitine, which was injected by a constant velocity pump and used to induce arrhythmias. The onset time and onset dosage of aconitine that induced ventricular premature contraction (VPC), ventricular tachycardia (VT) and ventricular fibrillation (VF) were measured.

### Drugs and reagents

Icariin (purity >97%) was obtained from Sigma Aldrich (Saint Louis, MO, USA). Collagenase type I and CsCl were purchased from Gibco (GIBCO TM, Invitrogen Co., Paisley, UK). BSA and HEPES were obtained from Roche (Basel, Switzerland), and the other chemicals were obtained from Sigma Aldrich (Saint Louis, MO, USA). Dimethyl sulfoxide (DMSO) was used to dissolve icariin to obtain a 1 mM stock solution. The final concentration of the DMSO added to the bath solution was less than 0.1%.

### Data analysis

Fitmaster (v2x32, HEKA) was used for data analysis, and the figures were plotted by Origin 8.0 (OriginLab Co., MA, USA). All data were expressed as the mean ± SD. Data pertaining to the I_NaT_ and I_CaL_ steady-state activation and steady-state inactivation relationships were fitted by the Boltzmann equation, *Y* = 1/{1+exp (*V*_*m*_−*V*_1/2_)/*k*}, where *V*_*m*_ is the membrane potential, *V*_1/2_ is the half-activation and half-inactivation potential, *k* is the slope factor, and *Y* is relative conductance (*G/G*_*max*_, steady-state activation) and relative current (*I/I*_*max*_, steady-state inactivation). The dose-response relationship curves for the effects of icariin on I_NaT_ and I_CaL_ were fitted to the Hill equation, (*I*_*control*_−*I*_*drug*_)/*I*_*drug*_ = *E*_*max*_/[1 + (*IC*_50_*/C*)^*n*^], where *I*_*control*_ and *I*_*drug*_ represent the amplitude of I_NaT_ and I_CaL_ obtained in the absence and presence of icariin, respectively, *E*_*max*_ is the maximum inhibition, *IC*_50_ is the concentration of icariin at which its half-maximum inhibitory effects are exerted, *C* is the concentration of icariin, and *n* is the Hill coefficient. Current density was calculated by dividing the current amplitude by the cell capacitance. The statistical significance of the differences between two groups was determined by Student's *t*-test, and mean comparisons among multiple groups were performed by one-way analysis of variance (ANOVA) followed by Bonferroni's test. *P* < 0.05 was considered significant.

## Results

**Figure 1:**
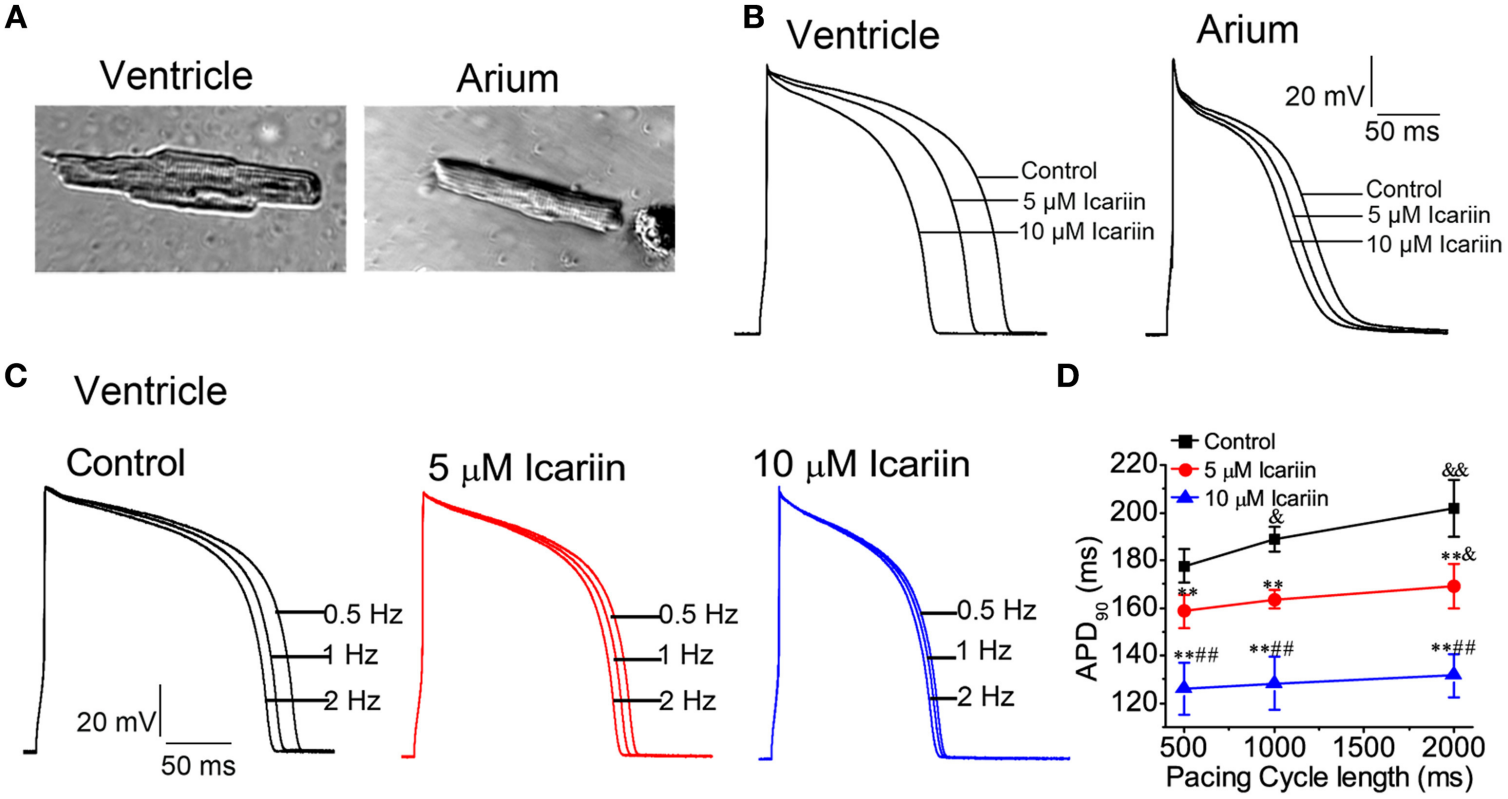
**Icariin attenuated APDs in a concentration- and rate-dependent manner in rabbit LVMs and LAMs. (A)**. Photomicrograph of a single LVM (left) and LAM (right). **(B)**. Effects of icariin (5 and 10 μM) on APs elicited at a stimulation frequency of 1 Hz in LVMs (left) and LAMs (right). **(C)**. Representative recordings of APs elicited at 0.5 Hz, 1 Hz and 2 Hz in the absence and presence of icariin (5 and 10 μM) in ventricular myocytes. **(D)**. Data pertaining to APD_90_ from 30 sequential curves were averaged. The averaged data for different pacing CLs are shown, *n* = 9 cells/4 rabbits. ^&^ and ^&&^*p* < 0.05 and 0.01 vs. a pacing CL of 500 ms; ^**^ *p* < 0.01 vs. control at the same pacing CL; ^##^*p* < 0.01 vs. 5 μM icariin at the same pacing CL.

Figure 1A shows the representative morphologies of a single isolated left ventricular myocyte (LVM, left) and left atrial myocyte (LAM, right). The rod-shaped LVM had glossy and smooth edges, as well as the typical transverse striations. The LAM was more slender than the LVM.

### Effects of icariin on action potentials

APs were consecutively recorded by 5-ms and 1.5-fold threshold current pulses at 1 Hz in the absence and presence of icariin. Icariin attenuated AP amplitude (APA) and the maximum upstroke velocity (V_max_), shortened action potential durations (APDs) at 50 and 90% repolarization (APD_50_ and APD_90_, respectively) in a concentration-dependent manner in LVMs and LAMs. However, icariin had no significant effects on resting membrane potential (RMP) at concentrations of 5 and 10 μM (Figure 1B; Table 1).

**Table 1:** **Effects of icariin on action potentials (APs) in rabbit LVMs and LAMs**.

	**Ventricle (*****n*** = **14 cells/7 rabbits)**			**Atrium (*****n*** = **14 cells/6 rabbits)**		
**Parameters**	**Control**	**5 μM icariin**	**10 μM icariin**	**Control**	**5 μM icariin**	**10 μM icariin**
RMP(mV)	−81 ± 2	−81 ± 3	−81 ± 5	−77 ± 3	−77 ± 4	−77 ± 6
APA(mV)	114 ± 5	111 ± 6	108 ± 9^*^	107 ± 7	104 ± 6	100 ± 5^*^
V_max_(V/s)	168 ± 10	146 ± 8^*^	131 ± 6^*^^†^	231 ± 10	222 ± 7	205 ± 13^*^^†^
APD_50_(ms)	158 ± 6	127 ± 5^*^	107 ± 3^*^^†^	103 ± 4	90 ± 9^*^	72 ± 7^*^^†^
APD_90_(ms)	188 ± 4	157 ± 3^*^	131 ± 4^*^^†^	135 ± 8	122 ± 12^*^	104 ± 11^*^^†^

*RP, resting membrane potential; APA, action potential amplitude; V_max_, maximum upstroke velocity; APD_50_, action potential duration at 50% repolarization; APD_90_, action potential duration at 90% repolarization*.

* p < 0.05 vs. control;

† *p < 0.05 vs. 5 μM icariin*.

In our study, 5 and 10 μM icariin attenuated the rate-dependence (RD) of the APDs (*n* = 9 cells/4 rabbits; Figures 1C,D) in LVMs by 10.5 ± 4.3% and 28.5 ± 7.2% at a pacing cycle length (CL) of 500 ms, by 13 ± 4.9% and 32.2 ± 7.4% at a pacing CL of 1,000 ms and by 16.5 ± 4.8% and 34.5 ± 6.4% at a pacing CL of 2,000 ms, respectively.

### Effects of icariin on cellular arrhythmias

**Figure 2:**
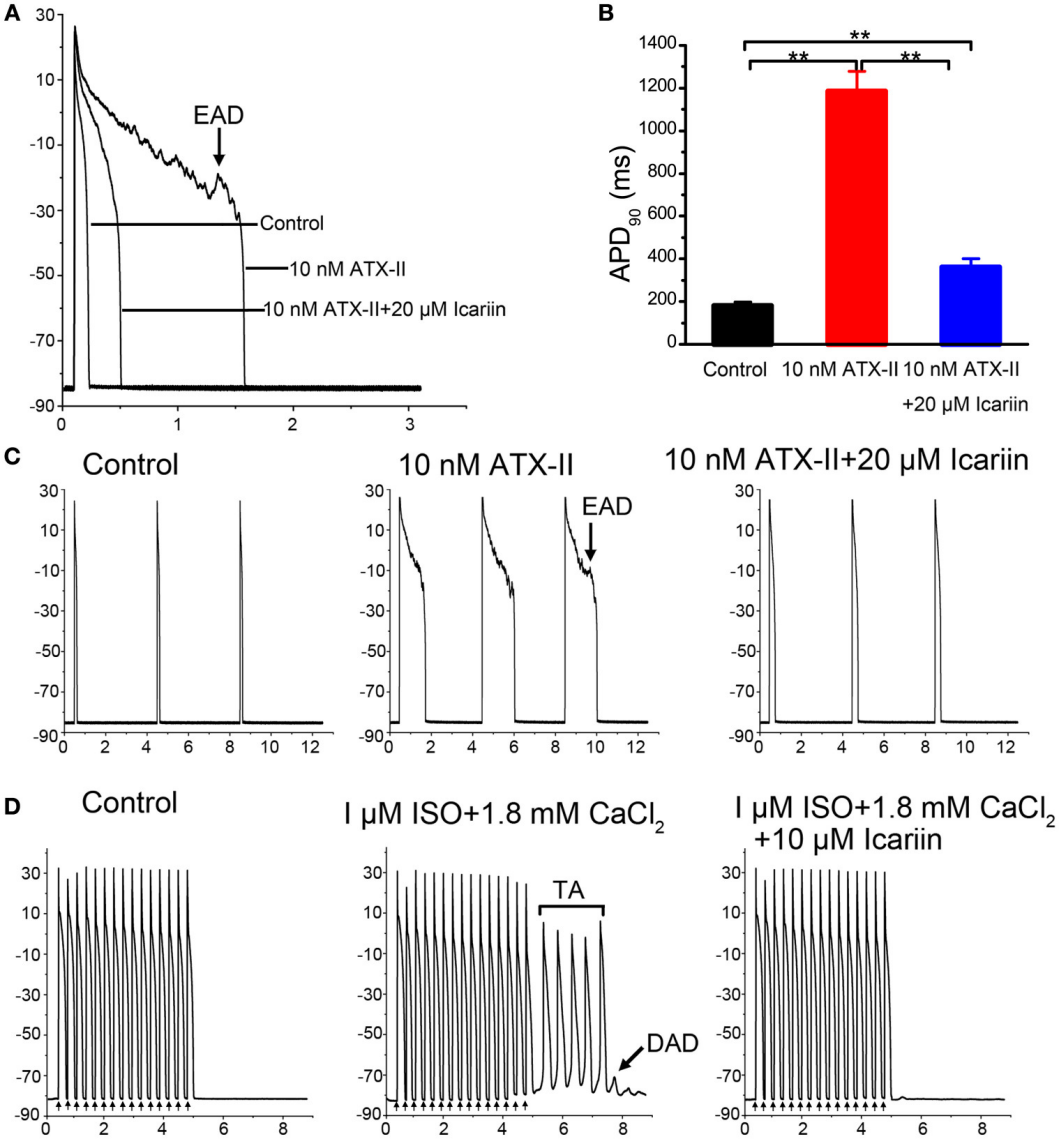
**Effects of icariin on EADs and DADs, as well as TAs, in LVMs. (A,C)**. Administration of 20 μM Icariin completely abolished the EADs and attenuated the AP prolongations induced by 10 nM ATX-II at a stimulation frequency of 0.25 Hz. Single beats or 3 consecutive beats are displayed in **(A,C)**, respectively. **(B)** Summary data for APD_90_ after sequential administration of 10 nM ATX-II and 20 μM icariin. ^**^p < 0.01. **(D)**. DADs and TAs were induced in LVMs by a baseline pacing CL of 9,000 ms and on top of that 15 beats with a stimulation frequency of 2.5 Hz after superfusion with ISO (1 μM) and a high extracellular calcium concentration ([Ca^2+^]_o_, 3.6 mM). Administration of 10 μM icariin significantly suppressed the DADs and completely abolished the TAs induced by ISO and calcium.

In the present study, we used 10 nM anemonia toxin II (ATX-II) and a stimulation frequency of 0.25 Hz to elicit early afterdepolarizations (EADs) in LVMs. ATX-II significantly lengthened the APD from 179.78 ± 18.64 ms to 1186.44 ± 93.13 ms and induced EADs in 7 of 10 cells (70%; *n* = 10 cells/5 rabbits; Figures 2A–C), and 20 μM icariin decreased the APs prolonged by ATX-II from 1186.44 ± 93.13 ms to 360.08 ± 41.95 ms and completely abolished the EADs induced by ATX-II in seven cells. In another group, to elicit delayed afterdepolarizations (DADs) and triggered activities (TAs) in LVMs, we added 1 μM isoproterenol (ISO) to the external solution and the extracellular calcium concentration was elevated to 3.6 mM following a baseline pacing CL of 9,000 ms and on top of that 15 beats with a stimulation frequency of 2.5 Hz. DADs were noted in 6 of 9 cells (3 rabbits; 66.7%), and TAs were noted in 3 of 9 cells (33.3%). Administration of 10 μM icariin significantly suppressed the ISO-induced DADs and completely abolished the ISO-induced TAs (Figure 2D).

### Effects of icariin on I_NaT_ and I_NaL_

**Figure 3:**
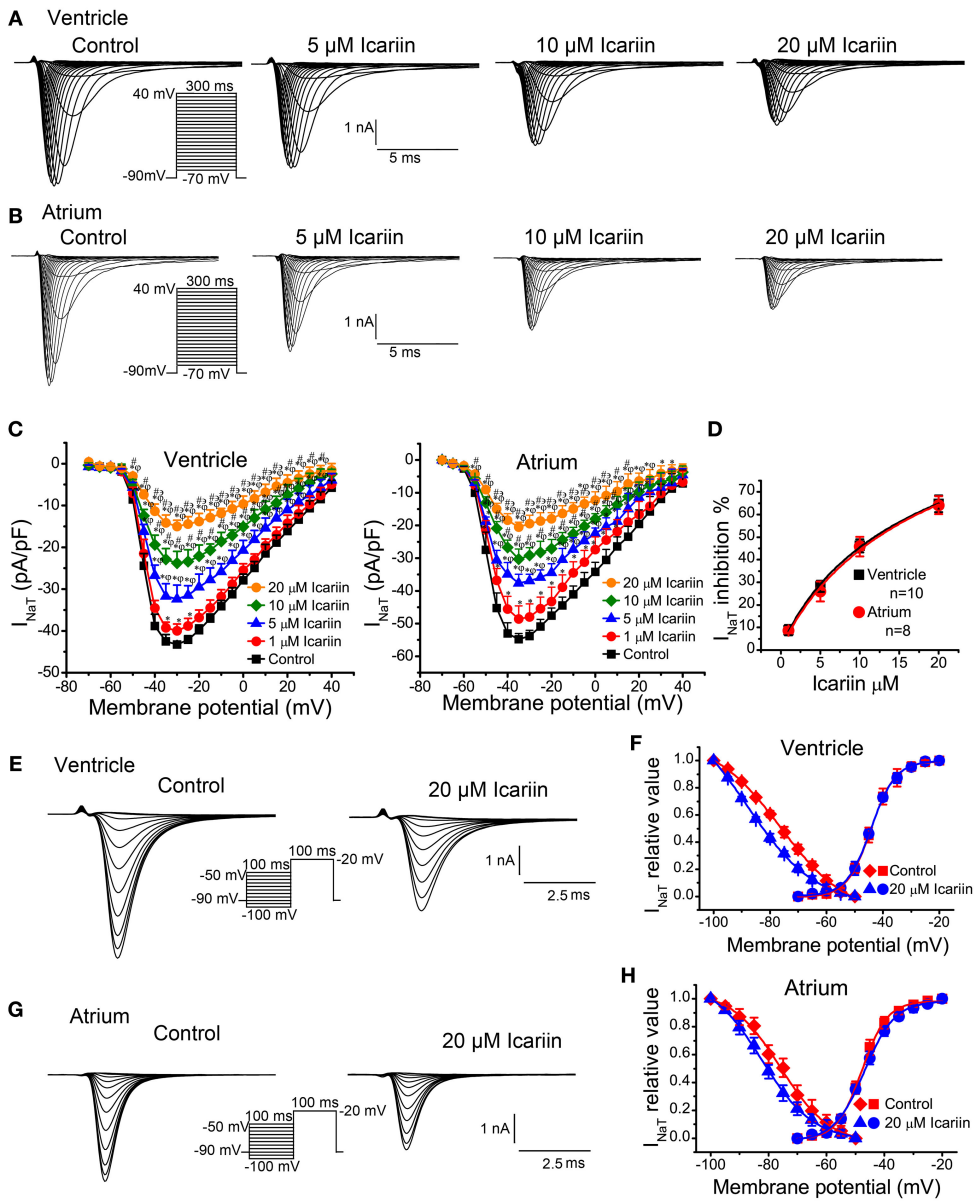
**Effects of icariin on I_**NaT**_ in LVMs and LAMs. (A,B)**. Representative recordings of I_NaT_ in LVMs **(A)** and LAMs **(B)** after sequential applications of 5, 10, and 20 μM icariin. **(C)**. Current-voltage relationship of I_NaT_ in LVMs (left; *n* = 10 cells/6 rabbits) and LAMs (right; *n* = 13 cells/5 rabbits) in the absence and presence of icariin. ^*^p < 0.05 vs. control; ^φ^*p* < 0.05 vs. 1 μM icariin; ^#^*p* < 0.05 vs. 5 μM icariin; ^϶^*p* < 0.05 vs. 10 μM icariin. **(D)**. The dose-response relationships illustrating icariin-induced decreases in I_NaT_ in LVMs and LAMs. Data were fitted by the Hill equation. **(E**,**G)**. Representative current recordings of I_NaT_ elicited according to the steady-state inactivation protocol in LVMs **(E)** and LAMs **(G)** in the absence and presence of 20 μM icariin. (**(F)**. Steady-state activation (*n* = 8 cells/4 rabbits) and steady-state inactivation (*n* = 8 cells/5 rabbits) curves of I_NaT_ in LVMs before and after icariin administration. (**(H)**. Steady-state activation (*n* = 8 cells/4 rabbits) and steady-state inactivation (*n* = 6 cells/3 rabbits) curves of I_NaT_ in LAMs before and after icariin administration.

When the effects of icariin on I_NaT_ reached a steady state (3 min), the next concentration of the drug could be added to the external recording solution. Icariin (1, 5, 10, and 20 μM) reduced I_NaT_ in a dose-dependent manner in LVMs and LAMs. Figures 3A,B show the representative recordings for I_NaT_ in LVMs and LAMs, respectively, and Figure 3C shows the corresponding current-voltage relationships in LVMs and LAMs. The IC_50_ values for I_NaT_ in LVMs and LAMs were 11.83 ± 0.92 μM (*n* = 10 cells/6 rabbits) and 12.28 ± 0.29 μM (*n* = 8 cells/4 rabbits; *p* > 0.05 LAMs vs. LVMs; Figure 3D), respectively. Figures 3E,G show typical current recordings, which were generated according to the steady-state inactivation protocol, in LVMs and LAMs. In the absence and presence of 20 μM icariin, the *V*_1/2_ values of the steady-state inactivation curves in LVMs were −85.47 ± 1.36 mV and −91.45 ± 1.48 mV (*n* = 8 cells/5 rabbits; *p* < 0.01 vs. control), respectively, with corresponding *k*-values of 8.48 ± 1.05 and 8.28 ± 0.76 (*n* = 8 cells/5 rabbits; *p* > 0.05 vs. control). Administration of 20 μM icariin shifted the *V*_1/2_ value of the steady-state inactivation curve in LAMs from −76.1 ± 1.52 mV to −82.28 ± 0.96 mV (*n* = 6 cells/3 rabbits; *p* < 0.01 vs. control), with *k*-values of 8.29 ± 1.64 and 8.72 ± 0.81 (*n* = 6 cells/3 rabbits; *p* > 0.05 vs. control). These results indicate that icariin induced a leftward (negative potential) shift of the steady-state inactivation curve of I_NaT_ in LVMs and LAMs (Figures 3F,H). However, it had no significant effects on the activation process in LVMs and LAMs (Figures 3F,H).

**Figure 4:**
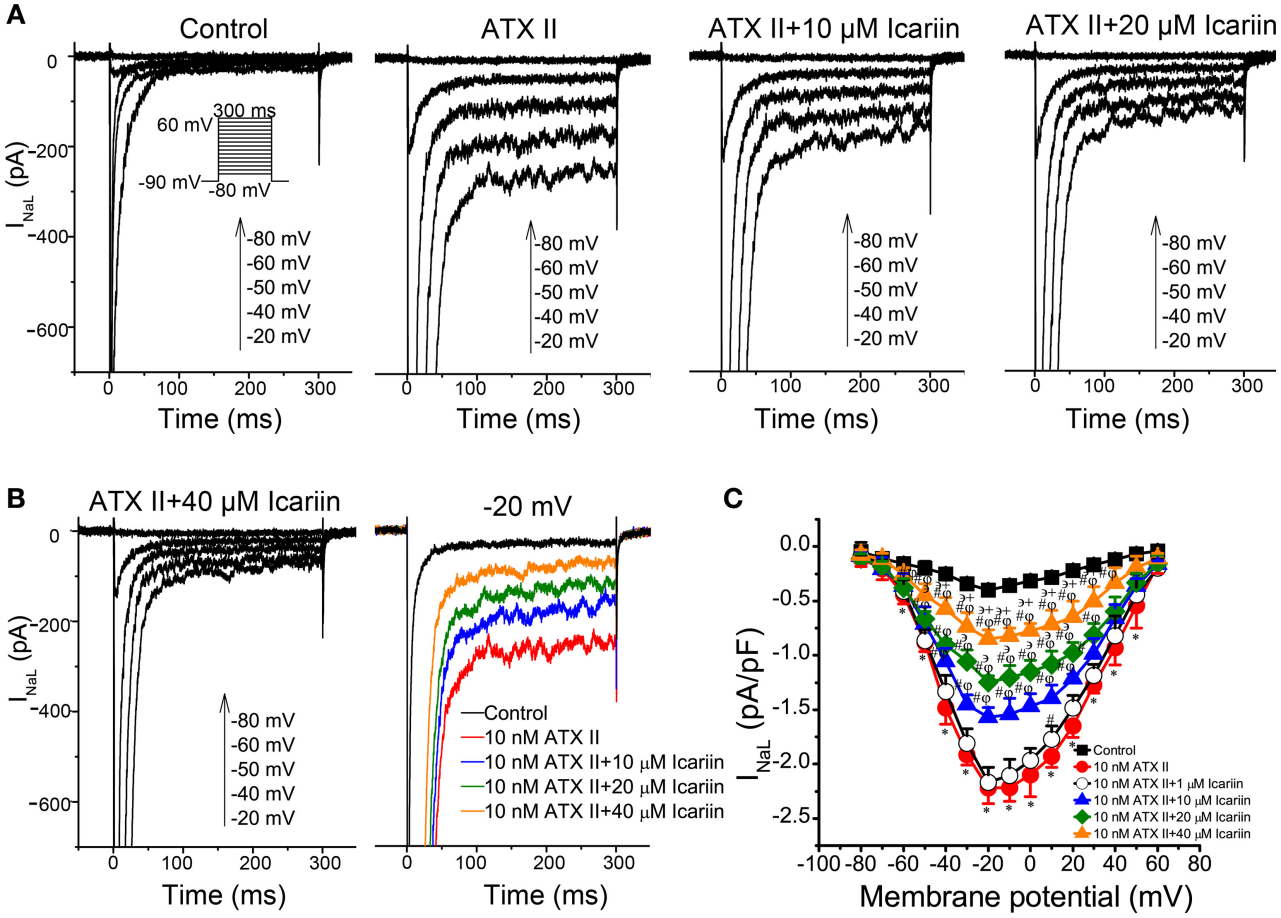
**Effects of icariin on ATX-II-induced increases in I_**NaL**_ in LVMs. (A)**. Representative current recordings of I_NaL_ in LVMs after sequential treatments with 10 nM ATX-II and 10, 20, and 40 μM icariin. (**B)**. Representative current recordings of I_NaL_ at −20 mV from **(A**,**C)**. The current-voltage relationship of I_NaL_ in LVMs, *n* = 6 cells/4 rabbits. ^*^p < 0.05 vs. control; ^#^*p* < 0.05 vs. 10 nM ATX-II; ^φ^*p* < 0.05 vs. 1 μM icariin; ^϶^*p* < 0.05 vs. 10 μM icariin; ^+^*p* < 0.05 vs. 20 μM icariin.

To identify I_NaL_, we recorded current before and after the application of 4 μM TTX using 300-ms depolarization pulses with potentials ranging from a HP of −90 mV to a potential of −20 mV. TTX (4 μM) had no significant effects on I_NaT_ but decreased the amplitude of I_NaL_ from −0.39 ± 0.004 pA/pF to 0.023 pA/pF (*n* = 6 cells/3 rabbits; *p* < 0.01 vs. control), indicating that the TTX-sensitive current was I_NaL_. Administration of 10 nM ATX-II significantly enhanced I_NaL_, an effect that was reversed by administration of 1, 10, 20, and 40 μM icariin (*n* = 6 cells/4 rabbits; Figures 4A,C). The percentage inhibitions by 1, 10, 20, and 40 μM icariin of ATX-II augmented I_NaL_ were 7.8 ± 1%, 29 ± 6.4%, 43.68 ± 5.6%, and 61.4 ± 5.7%. Figure 4B shows the representative current recordings of I_NaL_ at −20 mV that are shown in Figure 4A.

### Effects of icariin on I_CaL_

**Figure 5:**
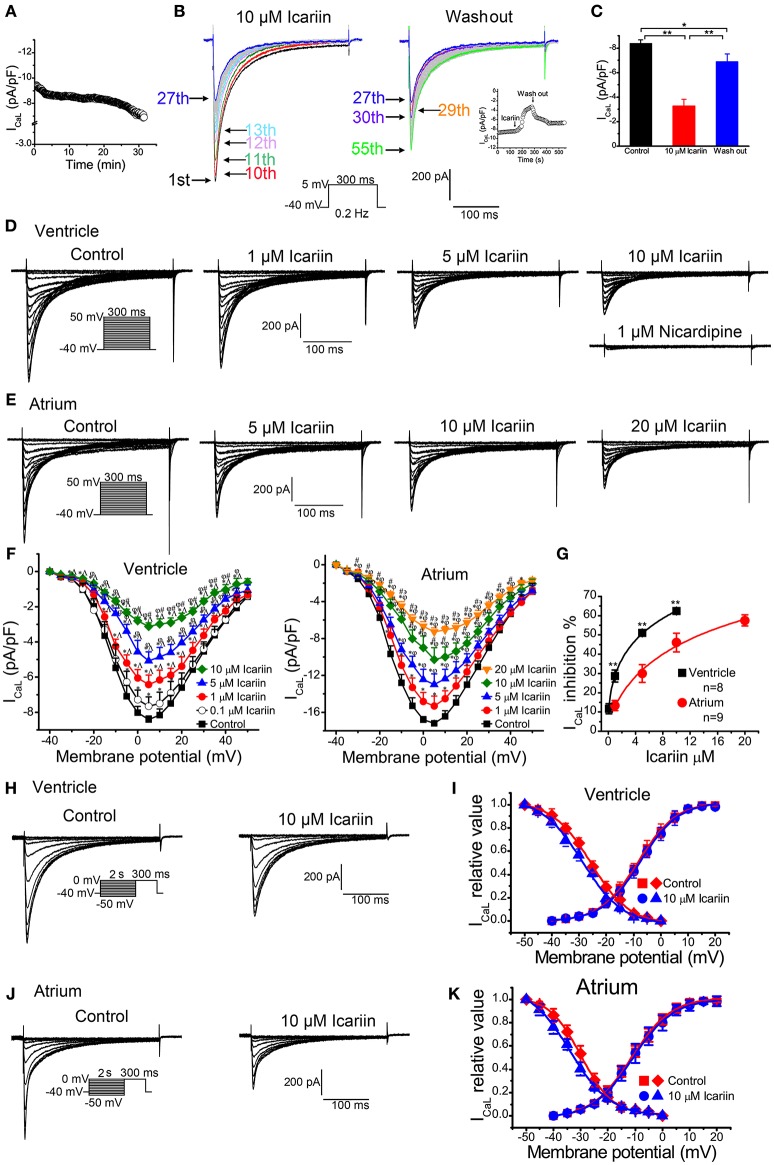
**Effects of icariin on I_**CaL**_ in LVMs and LAMs. (A)**. Time course of the I_CaL_ run-down phenomenon in LVMs under the control condition, *n* = 5 cells/2 rabbits. I_CaL_ were evoked by 300-ms depolarization pulses ranging from a holding potential of −40 mV to 5 mV at 0.2 Hz. **(B)**. Consecutive recordings of I_CaL_ evoked by a 300-ms depolarization from a holding potential of −40 mV to 5 mV at 0.2 Hz. The 1st sweep represents the control condition, the 2nd to 27th sweeps represent the period in which icariin exerted its effects on I_CaL_, and the 27th–55th sweeps indicate the period in which the effects of icariin on I_CaL_ were reversed. The inset represents the time course of the entire process, including the control condition, the icariin perfusion period, and the icariin wash-out period. The entire process was conducted during the stabilization period. (**(C)**. Summary data for the mean current densities of I_CaL_ in the control condition, the icariin perfusion period, and the icariin wash-out period, *n* = 10 cells/4 rabbits. ^*^ and ^**^p < 0.05 and 0.01. **(D)**. Representative current recordings of I_CaL_ in LVMs after sequential applications of 1, 5, and 10 μM icariin. **(E)**. Representative current recordings of I_CaL_ in LAMs in the absence and presence of 5, 10, and 20 μM icariin. **(F)**. Current-voltage relationship of I_CaL_ in LVMs (left; *n* = 13 cells/6 rabbits) and LAMs (right; *n* = 9 cells/5 rabbits) before and after the application of icariin. ^*^p < 0.05 vs. control; ^∧^*p* < 0.05 vs. 0.1 μM icariin; ^φ^*p* < 0.05 vs. 1 μM icariin; ^#^*p* < 0.05 vs. 5 μM icariin; ^϶^*p* < 0.05 vs. 10 μM icariin. **(G)**. The dose-response relationships illustrating icariin-induced decreases in I_CaL_ in LVMs and LAMs. ^*^ and ^**^ *p* < 0.05 and *p* < 0.01 LVMs vs. LAMs. Data were fitted by the Hill equation. **(H**,**J)**. Representative current recordings of I_CaL_evoked according to the steady-state inactivation protocol in LVMs **(H)** and LAMs **(J)** in the absence and presence of 10 μM icariin. **(I)**. Steady-state activation (*n* = 14 cells/7 rabbits) and steady-state inactivation (*n* = 10 cells/4 rabbits) curves of I_CaL_ in LVMs before and after icariin application. **(K)**. Steady-state activation (*n* = 12 cells/7 rabbits) and steady-state inactivation (*n* = 10 cells/6 rabbits) curves of I_CaL_ in LAMs before and after icariin application.

To elicit I_CaL_, we clamped LVMs at −40 mV and then depolarized the cells to +5 mV for 300 ms at 0.2 Hz. As shown in Figure 5A, the I_CaL_ run-down phenomenon lasted for approximately 5 min after membrane rupture in the control condition and then reached a steady state for 15 min (*n* = 5 cells/2 rabbits). I_CaL_ decreased by 8.5% during the this 5-min period. We performed a series of experiments on I_CaL_ during the stabilization period. To investigate the efficiency of the effects of icariin on I_CaL_ in LVMs, we recorded the current sequentially. As shown in Figure 5B, 10 μM icariin was added to the bath solution after the first (1st) current curve (control). I_CaL_ decreased rapidly between the tenth (10th) current curve (45 s after perfusion with icariin) and the thirteenth (13th) current curve (60 s after perfusion with icariin) and then decreased gradually until it reached a steady state (the twenty-seventh current curve). Icariin was washed out after 27th current curve (130 s after perfusion with icariin). I_CaL_ increased rapidly between the 27th current curve and the thirtieth (30th) current curve and then increased gradually until it reached its maximum value (82%) at the fifty-fifth current curve (270 s after perfusion with icariin). The summary data are shown in Figure 5C (*n* = 10 cells/4 rabbits). The above results indicate that icariin rapidly and reversibly inhibited I_CaL_ in LVMs.

When the effects of icariin on I_CaL_ reached a steady state (2.5 min), the next concentration of the drug could be added to the bath solution. Figure 5D shows the representative I_CaL_ recordings in LVMs after sequential treatments of 0.1, 1, 5, 10 μM icariin and 1 μM nicardipine. Icariin decreased I_CaL_ in a concentration-dependent manner in LVMs, with an IC_50_ of 4.78 ± 0.89 μM (*n* = 8 cells/4 rabbits; Figure 5G). Nicardipine (1 μM) almost completely inhibited I_CaL_ in LVMs in the presence of 10 μM icariin, indicating that I_CaL_ was the nicardipine-sensitive current. Figure 5E shows the representative I_CaL_ recordings in LAMs after sequential treatments of 1, 5, 10, and 20 μM icariin. Icariin reduced I_CaL_ in a dose-dependent manner in LAMs, with an IC_50_ of 13.43 ± 2.73 μM (*n* = 9 cells/5 rabbits; *p* < 0.01 vs. LVMs; Figure 5G). Figure 5F shows the I_CaL_ current-voltage relationships in LVMs (left, *n* = 13 cells/6 rabbits) and LAMs (right, *n* = 9 cells/5 rabbits). Icariin shifted the I_CaL_ steady-state inactivation curves to the left in LVMs and LAMs (Figures 5I,K). The *V*_1/2_ values before and after 10 μM icariin administration were shifted from −25.7 ± 1.01 mV and −29.96 ± 0.85 mV to −28.87 ± 2.18 mV (*n* = 10 cells/4 rabbits; *p* < 0.01 vs. control; Figures 5H,I) and −33.94 ± 1.33 mV (*n* = 10 cells/6 rabbits; *p* < 0.01 vs. control; Figures 5J,K), and the *k*-values were shifted from 7.16 ± 1.08 and 5.86 ± 0.82 to 7.52 ± 2.31 (*p* > 0.05 vs. control) and 7.4 ± 1.14 (*p* < 0.01 vs. control) in LVMs and LAMs, respectively. However, the drug has no significant effects on the activation process in these cells (Figures 5I,K).

### Effects of icariin on main potassium currents

**Figure 6:**
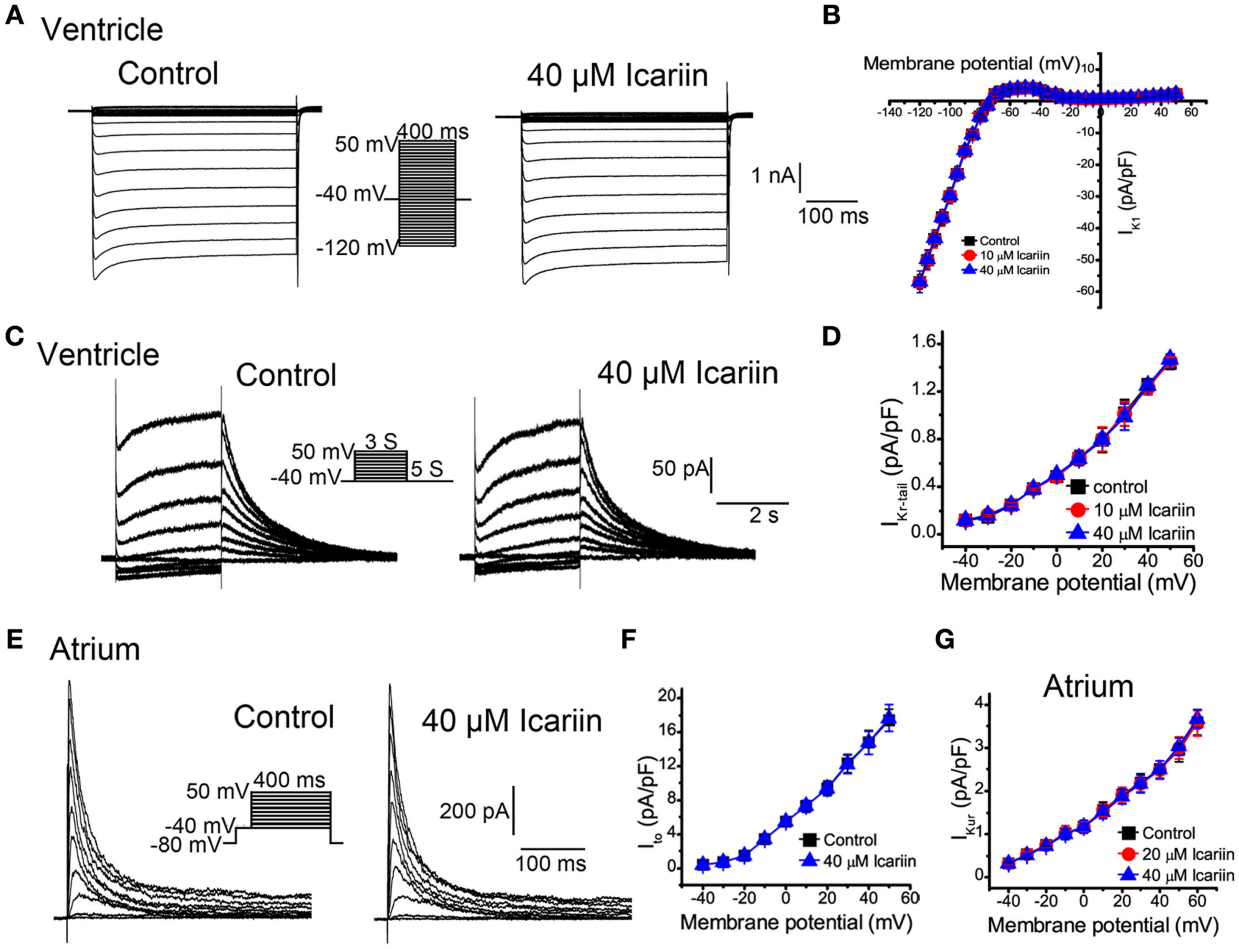
**Effects of icariin on the main potassium current in LVMs and LAMs. (A)**. Representative current recordings of I_K1_ in LVMs before and after 40 μM icariin application. **(B)**. The current-voltage relationship of I_K1_ in LVMs in the absence and presence of icariin, *n* = 18 cells/8 rabbits. **(C)**. Representative current recordings of I_Kr_ in LVMs before and after 40 μM icariin application. **(D)**. The current-voltage relationship of I_Kr_ in LVMs in the absence and presence of icariin, *n* = 12 cells/5 rabbits. **(E)**. Representative current recordings of I_to_ in LAMs before and after 40 μM icariin application. **(F)**. The current-voltage relationship of I_to_ in LAMs in the absence and presence of icariin, *n* = 15 cells/6 rabbits. **(G)**. The current-voltage relationship of I_Kur_ in LAMs before and after icariin application (20 an 40 μM), *n* = 15 cells/5 rabbits.

To elicit I_K1_ in LVMs, we clamped the cells at −40 mV (to inactivate their sodium channels) and depolarized them from −120 mV to +50 mV in 5-mV increments for 400 ms at 0.5 Hz. As shown in Figures 6A,B, icariin (10 and 40 μM) had no effect on I_K1_ (*n* = 18 cells/8 rabbits). I_Kr_ in LVMs was elicited using a 3-s depolarization pulse whose potential was increased from a HP of −40 mV to a potential of 50 mV in 10-mV increments before returning to a potential of −40 mV for 5 s. Only the I_Kr_ tail-current (I_Kr−tail_) was measured. Icariin (10 and 40 μM) had no significant effects on I_Kr−tail_ (*n* = 12 cells/5 rabbits; Figures 6C,D). I_to_ in LAMs was elicited by 400 depolarization voltage steps with potentials that were increased from −80 mV to +50 mV in 10-mV increments, followed by a conditional test in which −40 mV was administered for 100 ms to block sodium currents. Forty micrometer icariin had no significant effect on I_*to*_in LAMs (*n* = 15 cells/6 rabbits; Figures 6E,F). I_Kur_ in LAMs was elicited by an 80-ms prepulse whose potential was increased from a HP of −50 mV to a potential of 30 mV (to inactivate I_to_), followed by 140-ms test pulses with potentials that were increased from −40 mV to +60 mV in 10-mV increments—using a HP of −50 mV—after a 50-ms interval before returning to −30 mV. Figure 6G shows the I_Kur_ current-voltage relationship in LAMs in the absence and presence of icariin (20 and 40 μM). Icariin had no significant effect on I_Kur_ (*n* = 1 5 cells/5 rabbits).

### Effects of icariin on aconitine-induced arrhythmias

**Figure 7:**
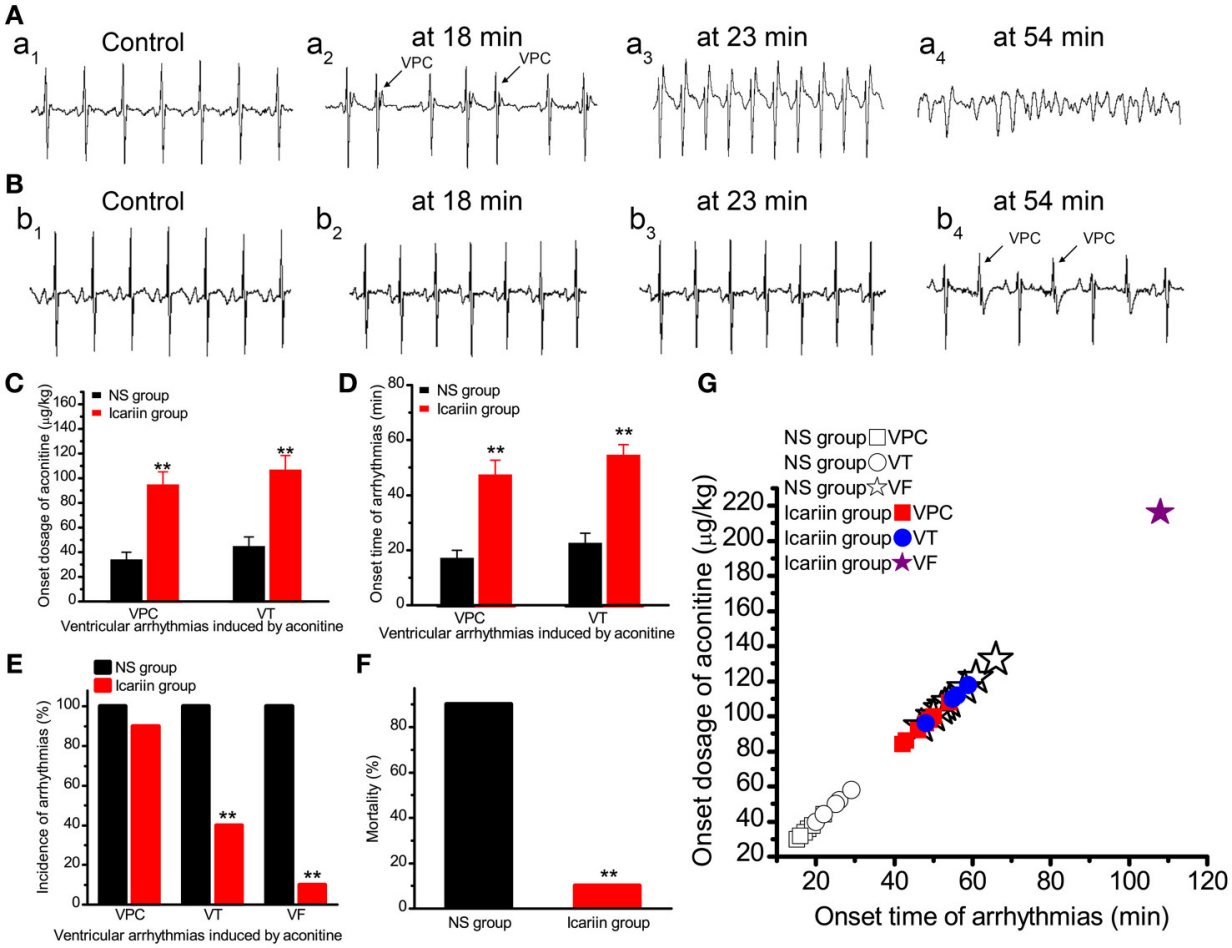
**Effects of icariin on aconitine-induced ventricular arrhythmias in whole rabbits. (A)**. Representative ECG recordings at different onset times of VPC (18 min), VT (23 min) and VF (54 min) in the NS group. **(B)**. Representative ECG recordings consistent with the times of events in **(A)** in the icariin group. **(C,D)**. Histograms show the onset dosage and onset time of aconitine-induced ventricular arrhythmias, respectively. ^**^p < 0.01. **(E)**. The incidence of VPC, VT, and VF induced by aconitine in the 2 groups. ^**^p < 0.01. **(F)**. Aconitine-induced mortality of rabbits in the 2 groups. ^**^p < 0.01. **(G)**. The onset time and onset dosage relationship of the 20 rabbits.

In the NS group, VPC, VT, and VF were observed in all 10 rabbits. In the icariin group, VPC, VT and VF occurred in 9, 4 and 1 of 10 rabbits, respectively. Compared with the NS group, icariin application prior to aconitine administration increased the onset time (Figures 7A,B,D) and onset dosage (Figure 7C). The administration of icariin attenuated the incidence of arrhythmias induced by aconitine (Figure 7E) and rabbit mortality (Figure 7F).

## Discussion

The main findings of the present study are as follows: (I) icariin reduced APA and V_max_ of APs, shortened APDs (APD_50_and APD_90_) in LVMs and LAMs (Table 1, Figure 1B). (II) Icariin decreased the RD of APD (Figures 1C,D) and significantly suppressed EADs and DADs and TAs induced by ATX-II or ISO and high [Ca^2+^]_o_, respectively, in LVMs (Figure 2). (III) Icariin decreased I_NaT_ in LVMs and LAMs (Figure 3) and attenuated the increases in I_NaL_ induced by ATX-II in a concentration dependent manner in LVMs (Figure 4). (IV) Icariin blocked I_CaL_ in a dose-dependent manner in LVMs and LAMs (Figure 5). Moreover, the inhibitory effects of icariin on I_CaL_ in LVMs were 2.8-fold stronger than those of icariin on the above current in LAMs. (V) Icariin had limited effects on I_K1_ and I_Kr_ in LVMs and on I_to_ and I_Kur_ in LAMs (Figure 6). (VI) Icariin inhibited aconitine-induced ventricular arrhythmias (Figure 7).

In this study, icariin decreased V_max_ of APs and shortened APDs (APD_50_ and APD_90_) in a concentration-dependent manner in LVMs and LAMs. The abovementioned decrease in APA and V_max_, which may be associated with the inhibitory effects of I_NaT_, can reduce conduction velocities, resulting in reentry blockade (Baba et al., 2005). Moreover, the APD shortening induced by icariin may be closely related to I_CaL_ inhibition because icariin does not affect I_K1_, I_Kr_, I_to_, and I_Kur_, which also play important roles in maintaining APD. Some drugs displays reverse rate dependence (RRD) of APD property, that is, the effect of a drug to prolong APD may be greater at slow than at fast heart rate, and vice versa. The findings of previous studies suggest that RRD of APD can be induced by enhancing I_CaL_ and inhibiting I_Kr_ or I_K1_(Bosch et al., 1998; Virag et al., 2009). RRD of APD enhancement leads to an increase in the cardiac transmural dispersion of the repolarization (Osadchii, 2013), which subsequently facilitates the occurrence of reentrant arrhythmias (Coronel et al., 2009; Maoz et al., 2014). In the present study, icariin attenuated I_CaL_ but had no effect on I_Kr_ or I_K1_, indicating that icariin might diminish or not produce RRD. These results suggest that icariin has increased antiarrhythmic efficiency compared with other drugs and that it is safer than its counterparts.

Sodium channels are known as the key targets of class I antiarrhythmic drugs. I_NaT_ is the main depolarization current in AP phase 0 and plays an important role in myocardial excitability and propagations (Goldin, 2002). In this study, icariin decreased the amplitude of I_NaT_, which caused a decrease in Na^+^ influx. Therefore, the results of this study indicate that icariin can relieve intracellular Na^+^ overload and exerts class I antiarrhythmic drug effects.

I_NaL_ is involved in the AP plateau phase (Kiyosue and Arita, 1989). A variety of pathological conditions, such as ischemia and hypoxia (Saint, 2006), cardiac hypertrophy and heart failure (Valdivia et al., 2005; Guo et al., 2014), can increase I_NaL_, resulting in an elevated intracellular sodium concentration ([Na^+^]_i_), as well as a subsequent increase in the intracellular Ca^2+^ concentration ([Ca^2+^]_i_) as a result of the activity of a reverse Na^+^/Ca^2+^ exchanger (NCX), leading to Ca^2+^ overload resulting in arrhythmia (Kihara and Morgan, 1991; Haigney et al., 1992; Yeh et al., 2008; Tang et al., 2012). On the other hand, increases in I_NaL_ can effectively lengthen the APD, resulting in EADs (Undrovinas et al., 1999). The authors of previous studies found that inhibiting I_NaL_ significantly prevented arrhythmias such as ventricular tachycardia and ventricular fibrillation (Pezhouman et al., 2014; Markandeya et al., 2016). Therefore, I_NaL_ is considered a new target for the treatment of arrhythmias (Undrovinas and Maltsev, 2008). In the present study, icariin reversed the increases in I_NaL_ induced by ATX-II (a known I_NaL_ opener), decreased I_CaL_, shortened the APD, and suppressed the EADs induced by ATX-II in LVMs. The percentage inhibitions by 1, 10, 20, and 40 μM icariin of ATX-II augmented I_NaL_ were 7.8 ± 1%, 29 ± 6.4%, 43.68 ± 5.6%, and 61.4 ± 5.7%. The percentage inhibitions by 3, 6, and 9 μM ranolazine of ATX II augmented I_NaL_ were 24 ± 6%, 44 ± 8%, and 62 ± 4% (Luo et al., 2013). The inhibitory effects of icariin on ATX-II augmented I_NaL_ is weaker than ranolazine (a known I_NaL_ blocker). Icariin can inhibit I_CaL_ and shorten APD, thus we concluded that icariin might inhibit ATX-II-induced arrhythmias by blocking I_NaL_ and I_CaL_.

I_CaL_ is one of the major inward currents in phase 2 of the AP and regulates Ca^2+^-related physiological processes (Benitah et al., 2010). Extracellular Ca^2+^ flows into cardiomyocytes mainly through L-type calcium channels and subsequently causes elevations in [Ca^2+^]_i_,which causes the sarcoplasmic reticulum to release large amounts of Ca^2+^ into the cytosol, a phenomenon known as Ca^2+^-induced Ca^2+^ release, which increases [Ca^2+^]_*i*_ further. A large number of studies have shown that various pathological conditions, including ischemia/reperfusion injury(de Diego et al., 2008) and heart failure (Casini et al., 2009), are associated with [Ca^2+^]_i_ abnormalities, especially intracellular Ca^2+^ overload, which plays a crucial role in the genesis of arrhythmias such as ventricular and atrial fibrillation (Kihara and Morgan, 1991; Yeh et al., 2008). Therefore, inhibiting I_CaL_ can facilitate [Ca^2+^]_*i*_reductions that suppress arrhythmias in the above pathological conditions. In this study, icariin decreased the amplitude of I_CaL_, which caused a decrease in Ca^2+^ influx. Therefore, icariin exerts class IV antiarrhythmic drug effects by inhibiting I_CaL_. DADs and TAs can be induced by [Ca^2+^]_i_ overload caused by the application of ISO and high [Ca^2+^]_o_ (Shutt et al., 2006; Sicouri et al., 2013). In the present study, icariin significantly suppressed DADs and TAs in LVMs, possibly by inhibiting I_CaL_. Moreover, the inhibition of I_CaL_ induced by icariin in LVMs was 2.8-fold stronger than that induced by icariin in LAMs. Thus, icariin shows a degree of ventricular selectivity with respect to its inhibitory effects on I_CaL_.

Elevations in [Ca^2+^]_i_ increase I_NaL_ by activating the CAMK II and PKC pathways (Ma et al., 2012; Wu et al., 2015). The increased I_NaL_ elevates [Na^+^]_i_, which increases [Ca^2+^]_i_ by activating a reverse NCX (Kihara and Morgan, 1991; Haigney et al., 1992; Yeh et al., 2008; Tang et al., 2012). The cellular response may cause or aggravate arrhythmias. In the present study, icariin inhibited both sodium currents (I_NaT_ and I_NaL_) and I_CaL_, which blocked the cellular response more effectively, indicating that icariin may be a more effective antiarrhythmic drug than established medications.

I_Kr_ is an important outward current in AP repolarization. Decreases in I_Kr_ lengthen the APD and lead to QT interval prolongation. A variety of noncardiovascular drugs can block I_Kr_, thereby inducing long QT syndrome and torsade de pointes (TdPs) (Viskin et al., 2003). For example, grepafloxacin, a quinolone antibiotic, was withdrawn from the American drug market because it blocked I_Kr_ significantly and caused excessive QT interval prolongation, resulting in TdPs (Anderson et al., 2001). Therefore, the authors of another study measured I_Kr_ antagonist potency to evaluate the proarrhythmic effects of new drugs (Kim et al., 2016) and found that it did not affect I_Kr_. In this study, icariin showed no effect on I_Kr_. Thus, we deemed the compound a safer drug than its established counterparts.

Aconitine, a specific sodium channel agonist, sustained activation of the sodium channels and induced intracellular Na^+^ accumulation leading to intracellular Ca^2+^ overload through NCX (Peper and Trautwein, 1967). Moreover, icariin can augment I_CaL_ directly causing intracellular Ca^2+^ overload, which may eventually result in arrhythmias (Zhou et al., 2013). In the present study, we found that icariin increased the onset time and onset dosage of aconitine-induced VPC, VT and VF in whole rabbits. It also decreased the incidence of aconitine-induced VT and VF, as well as mortality in rabbits. The above results indicate that icariin shows cardioprotective effects against aconitine-induced arrhythmias. The cardioprotective effects may be due to reduction of I_NaT_, I_NaL_ and I_CaL_.

## Conclusion

In summary, we found for the first time that icariin exerted class I and IV antiarrhythmic agent effects and moderately inhibited I_NaL_. Icariin inhibits aconitine-induced arrhythmias in whole rabbits. Icariin also suppressed EADs or DADs and TAs induced by ATX-II or ISO and high [Ca^2+^]_o_, respectively, by inhibiting I_NaT_, I_NaL_, and I_CaL_, but had no effect on I_K1_, I_Kr_, I_*to*_, and I_Kur_, especially I_Kr_, which may indicate that icariin is a safer drug than its counterparts. Thus, icariin may have promise as an agent used in the clinical treatment of arrhythmia.

## Author contributions

JM designed the research. WJ, MZ, and ZC performed the experiments. ZL, JH, PPZ, YT and PHZ analysis the data. WJ wrote the main text and prepared all of the figures. All authors reviewed and approved this manuscript.

### Conflict of interest statement

The authors declare that the research was conducted in the absence of any commercial or financial relationships that could be construed as a potential conflict of interest.

## Abbreviations

AP: action potential
LAM: left atrial myocyte
LVM: left ventricular myocyte
APD: action potential duration
APD_50_ and APD_90_: APD at 50 and 90% repolarization
V_max_: maximum upstroke velocity of AP
APA: AP amplitude
RMP: resting membrane potential
RD: rate dependence of the APD
RRD: reverse rate dependence of the APD
ATX-II: anemonia toxin II
EAD: early afterdepolarization
DAD: delayed afterdepolarization
TA: triggered activity
ISO: isoproterenol
[Ca^2+^]_o_: extracellular calcium concentration
I_NaT_: transient sodium current
I_CaL_: L-type calcium current
I_NaL_: late sodium current
I_K1_: inward rectifier potassium current
I_Kr_: rapid component of delayed rectifier potassium current
I_to_: transient outward potassium current
I_Kur_: ultra-rapid delayed rectifier potassium current
CL: cycle length
(VPC): ventricular premature contraction
(VT): ventricular tachycardia
(VF): ventricular fibrillation
